# Manipulating Crystallization for Simultaneous Improvement of Impact Strength and Heat Resistance of Plasticized Poly(l-lactic acid) and Poly(butylene succinate) Blends

**DOI:** 10.3390/polym13183066

**Published:** 2021-09-10

**Authors:** Todsapol Kajornprai, Supakij Suttiruengwong, Kalyanee Sirisinha

**Affiliations:** 1 Department of Chemistry, Faculty of Science, Mahidol University, Bangkok 10400, Thailand; kajornprai.t@gmail.com; 2 Department of Materials Science and Engineering, Faculty of Engineering and Industrial Technology, Silpakorn University, Sanamchandra Palace Campus, Nakhon Pathom 73000, Thailand; suttiruengwong_s@su.ac.th

**Keywords:** poly(lactic acid), poly(butylene succinate), impact strength, annealing, crystallization, heat resistance

## Abstract

Crystalline morphology and phase structure play a decisive role in determining the properties of polymer blends. In this research, biodegradable blends of poly(l-lactic acid) (PLLA) and poly(butylene succinate) (PBS) have been prepared by melt-extrusion and molded into specimens with rapid cooling. The crystalline morphology (e.g., crystallinity, crystal type and perfection) is manipulated by annealing the molded products from solid-state within a short time. This work emphasizes on the effects of annealing conditions on crystallization and properties of the blends, especially impact toughness and thermal stability. Phase-separation morphology with PBS dispersed particles smaller than 1 μm is created in the blends. The blend properties are successfully dictated by controlling the crystalline morphology. Increasing crystallinity alone does not ensure the enhancement of impact toughness. A great improvement of impact strength and heat resistance is achieved when the PLLA/PBS (80/20) blends are plasticized with 5% medium molecular-weight poly(ethylene glycol), and simultaneously heat-treated at a temperature close to the cold-crystallization of PLLA. The plasticized blend annealed at 92 °C for only 10 min exhibits ten-fold impact strength over the starting PLLA and slightly higher heat distortion temperature. The microscopic study demonstrates the fracture mechanism changes from crazing to shear yielding in this annealed sample.

## 1. Introduction

Over the past decade, poly(l-lactic acid) (PLLA) has attracted attention in many applications such as biodegradable medical devices, healthcare products, and single-use packaging. PLLA products have been produced by various forming processes such as solvent casting, injection molding, thermoforming, and extrusion. Recently the application of PLLA has expanded to three-dimensional (3D) printing where individually customized products are produced. Although having high stiffness, biocompatibility, and biodegradability, PLLA has a relatively low heat resistance and is considered brittle for some applications requiring high mechanical strength levels, high resistance to temperature, and good impact performance [1,2]. Therefore, simultaneous improvements of impact toughness and heat resistance of PLLA need to be made while preserving its biodegradability and sustainability.

Poly(butylene succinate) (PBS) is an aliphatic polyester with outstanding heat resistance and toughness [3,4]. PBS can biodegrade at lower temperatures (less than 35 °C) and is easily home compostable [3,4]. Hence, blending PLLA with PBS is suggested to obtain the fully biodegradable blend with improved thermal stability and toughness [5,6,7,8]. Even though remarkably enhanced tensile toughness and elongation can be easily obtained, [5,6] slight improvement in impact strength has been reported even at a high loading of PBS [9]. Many approaches have been taken to improve the impact toughness of the PLLA-based blends [10,11,12,13,14,15]. Most of the studies concentrated on applying an efficient modifier or compatibilizer in combination with reactive blending [11,12,13,14,15,16,17,18]. In the work of Zhang et al., a dramatic increase in elongation was observed in the PLLA containing 20% ethylene-methyl acrylate-glycidyl methacrylate terpolymer [12]. In the case of PLLA/PBS (70/30) blends, a significant improvement of elongation at break of 250% was achieved with the aid of adding linear PLLA-b-PBS-b-PLLA block copolymers, three-arm-branched copolymers, and the combination of both types [13]. Harada et al. used lysine triisocyanate as a reactive modifier to enhance the impact strength of PLLA/PBS (90/10) blends [15]. Up to now, researchers still look into a more complicated system for achieving a significant increase of impact strength in PLLA-based blends without sacrificing other important properties such as modulus and heat resistance. Unfortunately, the preparation of such reactive compatibilizers or reactive copolymers is not currently economically viable to be adopted by the industry for wide-scale production. Therefore, a more economical and effective method for obtaining high toughness PLLA of balanced stiffness and heat resistance is still needed.

Crystal structure and perfection as well as level of crystallinity play a vital role in the mechanical performance of the PLLA products. With increasing crystallinity, Young’s modulus of PLLA films increased but the elongation at break decreased [19]. Tensile strength showed similar behavior to modulus but decreased when large crystallites were formed [19]. In addition, a higher crystallinity implied a higher temperature resistance of the material. Basically, PLLA with D-content of less than 2% is classified as a semi-crystalline polymer. However, a high cooling rate used in traditional melt processing techniques, such as extrusion and injection molding, restricts its crystallization and results in a primarily amorphous material. Incorporating a highly active nucleating agent to the PLLA can effectively promote crystallization by providing nucleation sites and shortening the crystallization time [20,21]. Despite the successful enhancement of a crystallization rate of PLLA through the addition of nucleating agents, the PLLA-molded products of high crystallinity were hardly obtained under a fast mold cooling rate. The crystallinity of PLLA was found to increase through thermal annealing [22,23,24,25]. The PLLA sample that was annealed from melt to 65 °C and kept at this temperature for 1860 min (31 h) yielded the crystallinity of 43% while it reached 49% crystallinity after 30 min annealing at 80 °C [22]. Oyama reported a significant enhancement of the mechanical properties of PLLA by reactive blending with poly(ethylene-glycidyl methacrylate) (EGMA) and annealed the blend at 90 °C for 2.5 h. The crystallinity of annealed sample increased from 6% of pure polymer to 40% [23]. Besides the level of crystallinity, annealing temperature significantly dictated the structure of PLLA crystals (α- or α′ structure) formed in the molded products [24]. It is noted that even though the effects of annealing exerted on the mechanical properties of PLLA-based blends have been reported in the literature, most of the research work focused their studies on the properties of the blends after being annealed from melt or solid-state for a long period [22,23,26,27]. Only limited research works have been done on the PLLA/PBS blends intending to concurrently improve impact toughness and thermal resistance by controlling the crystallization of PLLA component through short-time annealing from solid-state, without the application of active nucleating agent and reactive compatibilizer.

In this study, attention was focused on improving both impact toughness and heat resistance of PLLA products by manipulating the crystallization morphology through simple blending and heat-treatment of the molded samples within a short time. In the work, PLLA/PBS (80/20) blend was selected as the model blend. PLLA and PBS were melt-mixed in a twin-screw extruder and molded into testing specimens under controlled conditions. The molded samples were rapidly cooled to freeze the phase morphology and prohibit the melt-crystallization of the PLLA matrix. The crystallization morphology of PLLA in the blends was tailored by performing a post-process annealing of the molded products at selected temperatures for 10, 20, and 30 min at maximum. The influences of medium molecular-weight poly(ethylene glycol) (PEG8000) on the crystallization and mechanical properties of the PLLA/PBS blends were addressed.

## 2. Materials and Methods

### 2.1. Materials

PLLA (4032D, NatureWorks LLC, Minnetonka, MN, USA) with high stereoregularity (less than 2% d-isomer lactide) was used as the matrix polymer. It has a glass transition (*T_g_*) at 59 °C and melting temperature (*T_m_*) at a high temperature of 167 °C, as determined by differential scanning calorimeter (DSC). It exhibits a melt flow index (MFI) of 7 g.10 min^−1^ (210 °C, 2.16 kg). BioPBS (FZ91PD) was from PTT MCC Biochem Co. Ltd. (Bangkok, Thailand), with *T_m_* of 114 °C and MFI (190 °C, 2.16 kg) of 5 g.10 min^−1^. PEG with an average molecular-weight of approximately 8000 g.mol^−1^ and *T_m_* of 64 °C was purchased from Aldrich Chemical Co., Milwaukee, WI, USA).

### 2.2. Blend Preparation

PLLA/PBS (80/20) blends and plasticized blends containing 5% PEG were prepared by melt mixing in a co-rotating twin-screw extruder (Prism TSE16, Staffordshire, UK) using a screw speed of 100 rpm and temperatures in the range of 150–190 °C. Before compounding, the PLLA and PBS pellets were dried in a vacuum oven at 80 °C for 4 h, whereas PEG powder was dried at 45 °C for 6 h.

### 2.3. Annealing Procedure

Samples to be used for the annealing experiment were obtained by compression-molding the granulates at 180 °C and fast cooling from the molding temperature to ambient temperature at a cooling rate of 100 °C.min^−1^. The molded samples were then annealed at different temperatures of 75, 92, and 152 °C to obtain samples of different crystalline morphology. The temperature of 75 °C represents the temperature that is fifteen degrees higher than the glass transition temperature of PLLA. The temperature of 92 °C is the temperature in the cold-crystallization region of PLLA while the temperature of 152 °C is forty degrees higher than the melting of PBS and close to the melting of PLLA. In this study, the maximum heat treatment time was kept within 30 min to be easily applied to the industry. After completing the heat treatment process, the annealed specimens were cooled down to the ambient temperature at a cooling rate of 100 °C.min^−1^.

### 2.4. Characterizations and Testing

The crystallization behaviors of PLLA, PLLA/PBS blends, and plasticized blend samples were investigated using a DSC Q200 differential scanning calorimeter (TA Instruments, New Castle, DE, USA). The samples of approximately 10 mg were sealed in an aluminum pan and then heated to 200 °C in a dry nitrogen atmosphere under a heating rate of 10 °C.min^−1^. The degree of crystallinity (*X_c_*) of the PLLA component in the blends was calculated from the measured enthalpies of melting (Δ*H_m_*) and cold-crystallization (Δ*H_cc_*) using the following equation (Equation (1)).

(1) $$X_{c}\left(\%\right)\;=\;\frac{\Delta H_{m}-\Delta H_{cc}}{w\cdot \Delta H_{m}^{0}}\times 100$$

where *w* is the weight fraction of PLLA in the blend. Here, the value of Δ*H_m_^0^* is defined as the melting enthalpy of a 100% crystalline poly(lactic acid) which was taken to be 93.0 J.g^−1^ from the literature [28].

The phase morphology of PLLA/PBS blends with and without PEG was observed through a field emission scanning electron microscope (FESEM Hitachi SU8000, Tokyo, Japan) with an accelerating voltage of 5 kV. The cryo-fractured surface was prepared by freezing and fracturing the samples in liquid nitrogen. The specimens were sputter-coated with a thin layer of platinum-palladium to prevent electrostatic charge build-up before observation. The morphological parameters were then analyzed from the FESEM micrographs using Image-Pro Plus 6.0 software. For each formulation, at least 300 particles from several micrographs were measured. The number (*d_n_*) and weight (*d_w_*) average sizes of the dispersed particles as well as the particle size distribution (*δ*) were calculated using the following equations [29].

(2) $$d_{n}=\frac{\sum_{i=1}^{N}n_{i}d_{i}}{\sum_{i=1}^{N}n_{i}}$$

(3) $$d_{w}=\frac{\sum_{i=1}^{N}n_{i}d_{i}^{2}}{\sum_{i=1}^{N}n_{i}d_{i}}$$

(4) $$\ln\sigma =\sqrt{\frac{\sum_{i=1}^{N}n_{i}{\left(\ln d_{i}-\ln d\right)}^{2}}{\sum_{i=1}^{N}n_{i}}}$$

Tensile testing was performed using an Instron Model 5566 Universal Tensile tester (Canton, MA, USA) under a crosshead speed of 50 mm.min^−1^ at ambient temperature. At least five test specimens for each blend formulation were used. The correlation between stress and strain was studied. Young’s modulus, tensile strength, and elongation at break were determined. The impact strength (in kJ.m^−2^) was determined by a notched Izod impact test with a 4.0 J pendulum at room temperature. The test specimens were in the form of a rectangular bar with a thickness of 3 mm.

Heat distortion temperature (HDT) testing was performed according to ASTM D648. The samples were immersed in a silicone oil bath at 40 °C for 3 min, then a constant load of 0.46 MPa was applied. The temperature was increased at 2 ± 0.2 °C.min^−1^. The HDT value is derived from the temperature of the heat-transfer medium when the specimen has deflected by 0.25 mm.

## 3. Results and Discussion

### 3.1. Stress-Strain Behaviors

**Figure 1:**
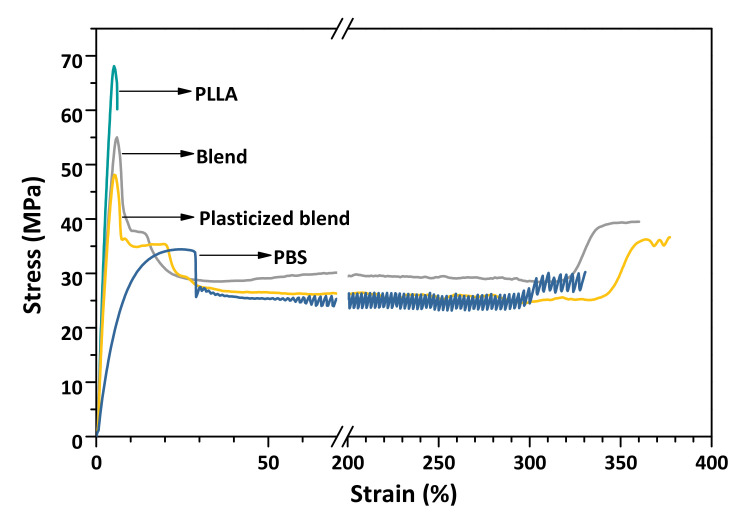
Stress–strain curves of PLLA, PBS, PLLA/PBS (80/20) blend, and plasticized blend containing 5% PEG.

The PLLA molded specimen is hard and very brittle, showing the failure at a low strain with the elongation at break of only 10%; in contrast, the PBS sample displays a yield point maximum in the stress–strain curve followed by cold-drawing and terminal increase in the stress before failure as shown in Figure 1. A non-uniform necking with stress fluctuation occurs in the cold-drawing region, accompanied by an apparent periodic formation of transparent and opaque banding perpendicular to the deformation direction. PBS exhibits a great extent of deformation with the elongation at break of approximately 300%. The addition of 20% PBS is very effective for overcoming the brittleness of PLLA, resulting in a significant increase in elongation at break. It is evident in Table 1 that the presence of PBS leads to a decrease in stiffness and strength, as observed in the Young’s modulus of 1.78 GPa and tensile strength of 55.69 MPa. The changes in tensile properties are more pronounced when PEG was present in the blend formulation (plasticized blend). For all studied systems, significantly decreased elongation at break are resulted after thermal treatment. Annealing temperature has more impact on the elongation than strength and modulus.

### 3.2. Impact Resistance

**Figure 2:**
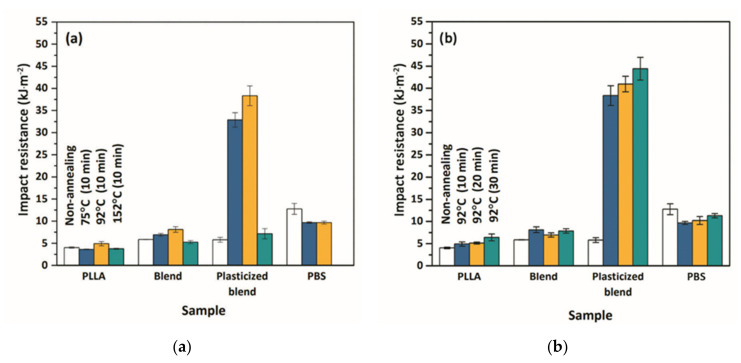
Effect of annealing temperatures (**a**), and duration (**b**) on notched Izod impact resistance.

In the as-molded specimens, the effects of PBS and PEG on the impact strength are slight (Table 1). The impact strength of PLLA improves slightly from 4.02 kJ.m^−2^ to 5.85 and 6.75 kJ.m^−2^ in the blend and plasticized blend, respectively. Interestingly, Figure 2 demonstrates a dramatic increase in the impact strength of the plasticized blends after thermal treatment. Remarkably high resistance to impact load is achieved in the samples annealed at 92 °C: the plasticized blend annealed at 92 °C for only 10 min reveals a high impact resistance of 38.34 kJ.m^−2^ which is five times higher than that of the as-molded sample and nearly ten-fold that of the parent PLLA (Figure 2a). Further increase in the impact strength to 44.39 kJ.m^−2^ is gained by annealing the samples for a longer period of 30 min (Figure 2b). The negative effect on impact strength is observed when performing the heat treatment at 152 °C. The impact strength of the plasticized blend drastically drops to 7.16 kJ.m^−2^. At the temperature of 152 °C which is much higher than the melting of PEG (*T_m_* of 64 °C) and PBS (*T_m_* of 114 °C), the loss of entanglement densities between the PLLA and PBS phases in the blend possibly occurs and thus causes a reduced efficiency to bear the load applied. A high percentage of crystallinity of the blend (54.6%) and plasticized blend (55.1%) after annealing at 152 °C (10 min) could not result in an enhancement of impact strength.

Several parameters have been known to affect the impact strength and toughening mechanism in PLLA blends [2,30,31,32,33]. These include elastomer or impact modifier content, particle size of the dispersed component, and interfacial adhesion between the dispersed particles and the matrix, etc. Indeed, these parameters are interrelated; for example, in rubber-modified polymer, the rubber particle size is a function of rubber content [30] and the diameter of dispersed particles in the blend may alter upon annealing from melt state [31]. In this study, no reactive compatibilizer was used, and hence no chemical interaction between phases is expected. In addition, the annealing experiment was conducted on the molded specimens in which their phase morphology has been carefully controlled during mixing and molding processes. Therefore, the annealing process used in this study (annealed the samples from solid-state) should barely affect the phase morphology of the molded samples. Thus, the interrelated effects of phase morphology and crystalline morphology could be deducted to some extent. This would allow for an understanding of how phase morphology and crystalline morphology influence the impact toughness and heat resistance of the PLLA/PBS blends.

### 3.3. Discussion on Impact Performance and Crystalline Morphology

To understand the effect of crystalline morphology on the impact performance, the relationship between crystallinity of PLLA matrix and impact resistance of the samples annealed at 75 and 92 °C is focused on. The melting and cold-crystallization enthalpies of the PLLA component were carefully analyzed, and the level of crystallinity of PLLA matrix in each formulation was calculated. The DSC thermograms of all samples have been given in our previous report and, therefore, are not shown here [24].

**Figure 3:**
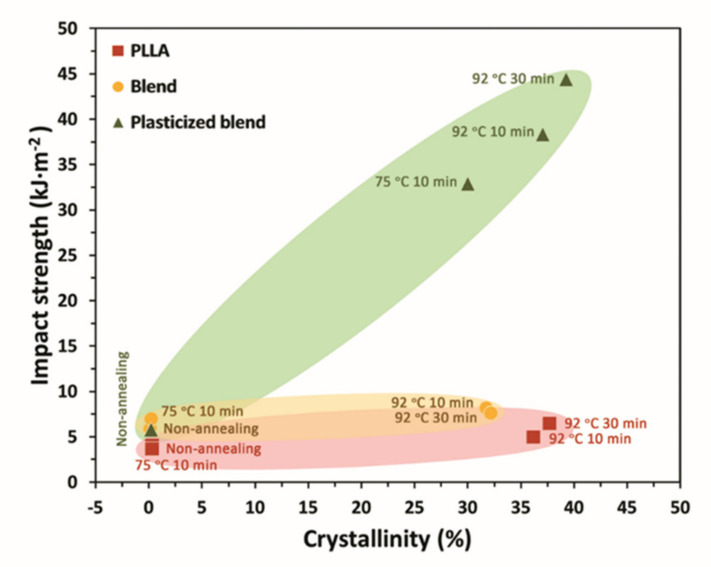
Relationship between crystallinity and notched Izod impact strength.

Figure 3 shows the plot of impact strength as a function of crystallinity. As expected, the as-molded PLLA specimen has a very low crystallinity. As a result, this sample cannot respond without rupture to rapidly applied impact loads and exhibits an impact strength of only 4.02 kJ.m^−2^. The PLLA/PBS (80/20) blend cannot achieve the anticipated improvement in the impact properties by either adding PEG plasticizer or performing heat treatment. Although increased crystallinity by thermal annealing from solid-state is possible, the formation of a highly crystalline PLLA-based blend is difficult to obtain. The resistance to impact load is gained only after the PLLA/PBS blends were plasticized with PEG and simultaneously heat-treated under the appropriate conditions.

The sample of 30% crystallinity is gained after annealing the plasticized blend for only 10 min at 75 °C, indicating that the crystallization of PLLA from solid-state is much easier in the presence of PEG. In this sample, a remarkably increased impact strength of 32.89 kJ·m^−2^ is obtained. The higher crystallinity of approximately 40% is achieved in the plasticized samples annealed at 92 °C for 30 min. As the crystallinity of the PLLA matrix increases, the impact strength increases systematically. Even though the increased crystallinity of the PLLA component could increase the impact resistance, the magnitude of impact improvement in the plasticized sample after annealing is considerably much greater than that of the annealed blend (without PEG). This suggests a crucial effect of PEG on the crystallization of PLLA in the systems. It is well-known that PLLA exhibits remarkably complex crystalline morphology [34,35,36]. Even at the same crystallinity, different effects on the performance may be obtained due to the different lamellar organizations [36]. Our recent study has shown the profound effects of annealing temperature on the crystal structure of PLLA [24]. The low-order α′- structure of PLLA was formed in the as-molded samples and all the PLLA/PBS blends annealed at the temperatures below the melting of PBS (at 75 and 92 °C). Only the pure PLLA sample showed the high order α-structure after being annealed at 152 °C for 10 min [24]. The crystal structure of PLLA component (α′-crystal) in the plasticized samples did not alter but crystal packing and perfection improved as increasing the time of heat treatment [24]. In this way, a significant enhancement of impact resistance in the plasticized blend annealed at 92 °C for 30 min is believed to be associated with the increased matrix crystallinity and the improved crystal packing and perfection in the system.

### 3.4. Discussion on Impact Performance and Phase Morphology

Besides the crystalline morphology of polymer matrix, the phase morphology of polymer blend is one of the influencing factors dictating the impact performance of polymer blends. Herein, the phase morphology of studied PLLA-based blends is investigated.

**Figure 4:**
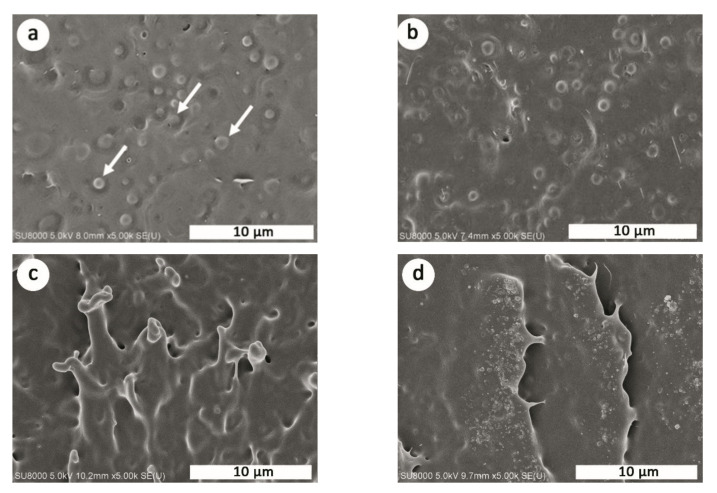
Cryogenic fractured surfaces of PLLA/PBS (80/20) blend (**a**) and plasticized blend (**b**), impact fractured surfaces of plasticized 80/20 blend annealed at 92 °C for 10 min (**c**), and of the non-annealed counterpart (**d**).

The phase-separation morphology is seen in the blend and plasticized blend samples in which the spherical PBS particles are dispersed evenly in the continuous PLLA matrix. An example of the phase-separation morphology is shown in Figure 4a. The diameter of the PBS particles in the 80/20 blends ranges from 0.75 to 1.25 μm, with the number average diameter (*d_n_*) of 0.93 μm, weight average diameter (*d_w_*) of 0.96 μm, and particle size distribution parameter (*δ*) of 1.2. For the plasticized blend containing PEG, the PBS domain sizes slightly reduce with the *d_n_*, *d_w_*, and *δ* values of 0.80 μm, 0.82 μm, and 1.2, respectively (Figure 4b). Similar findings were found in the previous study of Pivsa-Art et al. in which the addition of PEG resulted in smaller particles of PBS, confirming the role of PEG as a compatibilizer between PLA and PBS phases [37]. All annealed samples of this study also have the same dispersion state, the morphology of well-dispersed PBS particles. The PBS particles in the samples after annealing are slightly smaller than the non-annealed counterpart. For example, the plasticized blend after annealing at 92 °C for 10 min exhibits the *d_n_*, *d_w_*, and *δ* values of 0.73 μm, 0.76 μm, and 1.2, respectively. The treatment time used in this study (within 30 min) has an insignificant effect on those morphological parameters. In the literature, remarkable impact resistance of about 72 kJ.m^−2^ was obtained in the reactive blends of low molecular weight PLLA and poly(ethylene-co-glycidyl methacrylate) (EGMA) after annealing at 90 °C for 2.5 h [23]. With the aid of reactive compatibilizer and core-shell impact modifier, the impact resistance of PLLA/PC blend increased from 4.6 to 31.6 kJ.m^−2^ after applying a heat treatment at 120 °C for 6 h [38]. Great achievement in impact resistance in those systems was mainly due to an improved interfacial adhesion between phases in the blends brought about by the addition of reactive modifiers, and a long period of annealing. In this study, no reactive agents have been used. However, the PEG of this study with its medium molecular-weight of 8000 acts as a plasticizer promoting the rearrangement of polymer chains into a more order manner and plays the role of compatibilizer enhancing the compatibility between the PLLA and PBS phases in the blends. In other words, a significant enhancement of impact resistance in the plasticized samples annealed at 92 °C is owing to a combined effect of increased crystalline perfection and compatibility in the system promoted by PEG.

Generally, the polymer fracture process is dominated by two main mechanisms; crazing in the case of glassy amorphous matrix (e.g., poly(styrene), PS) and shear yielding in the case of semi-crystalline polymer (e.g., poly(propylene), PP). More importantly, shear yielding is a much more effective energy dissipation mechanism compared to crazing. Furthermore, toughening becomes easier to achieve with the increase of matrix crystallinity. For certain polymers, brittle polymers, in particular, there seems to be an optimum particle size for maximum toughness. For PS with a typical notched Izod impact strength of 21 J.m^−1^, the optimum rubber diameter of 2.5 μm was reported [39]. For PLLA-based blends, the optimum particle size was predicted using Wu’s relationship [39,40] and the calculated values in the range of 0.16–0.31 μm have been reported [41]. Although the sizes of dispersed PBS particles in our plasticized blends (in the range of 0.73–0.82 μm) are slightly larger than the predicted ones, the shear yielding mechanisms can be triggered as seen in Figure 4c, and this gives rise to a significant enhancement of impact toughness in this sample. Without heat treatment, a plasticized sample shows only a rough surface with some crests as shown in Figure 4d.

### 3.5. Overall Mechanical and Thermal Properties of PLLA-Based Blends

**Figure 5:**
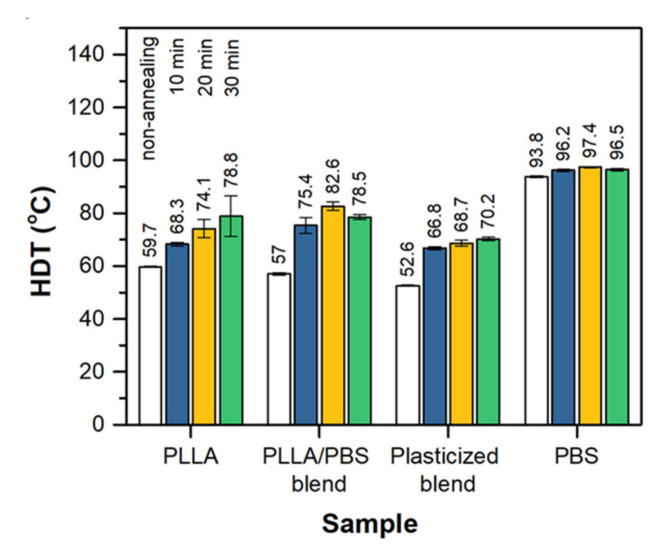
Heat distortion temperature (HDT) results. (Annealing temperature 92 °C).

Recalling that the macroscopic changes of mechanical properties depend strongly on the microstructure of a material. In this study, annealing is applied to control the crystal morphology of the blends. The favorable annealing condition for the PLLA-based blend is at 92 °C for the duration of only 10 min. Under that condition, the plasticized blend after applying heat treatment exhibits a high impact resistance of 38.34 kJ.m^−2^ which is ten-fold over that of the starting PLLA. In this formulation, the tensile modulus of 1.59 GPa and strength of 50.17 MPa are observed. Several researchers attempted to improve the toughness of PLLA-based blend by performing a thermal treatment. Unfortunately, great improvement in impact toughness is hardly obtained. For the PLLA/PC (70/30) blend, no improvement in impact strength was obtained even after thermal annealing at 120 °C for 6 h [42]. In the case of the blend with PBAT (70/30), the elongation at break dramatically reduced and also the impact resistance decreased from 19.03 to 12.11 kJ.m^−1^ after annealing at 100 °C for 8 h [43]. This is in contrast to the effect on HDT of the blends where the increased HDT values are mostly observed in the annealed samples [42,43]. The HDT of PLLA/PBAT blends increased from 50 to 80 °C after annealing at 100 °C for 24 h [43]. In our study, the starting PLLA exhibits an HDT of 59.7 °C (Figure 5). On the contrary to PLLA, PBS exhibits excellent heat resistance of 93.8 °C. It is expected that the heat resistance of PLLA would be increased by the presence of PBS in the blend. Unfortunately, the experimental results are not as expected. The HDT of 80/20 blend is even lower than the property of the main PLLA. Ostrowska et al. reported similar findings that the HDT values in the range of 56–59 °C were found for the blends with PBS content from 10–90% [9]. Plasticization of PLLA by PEG reduces the HDT of the material considerably. The thermal resistance of the PLLA and all blend samples is improved by performing thermal treatment of the samples at 92 °C. The longer the treatment time, the higher the HDT values. In this study, the blend annealed at 92 °C for 10 min exhibits the HDT value of 75.4 °C that is eighteen degrees higher than the non-annealed counterpart.

**Figure 6:**
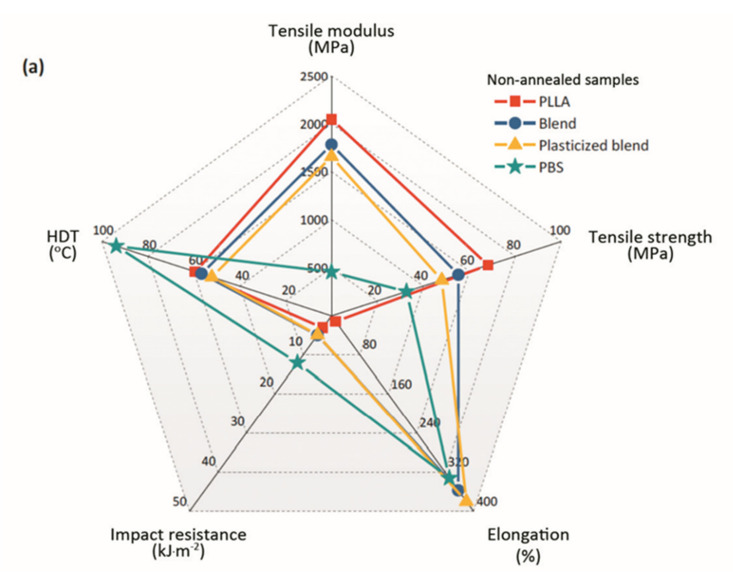

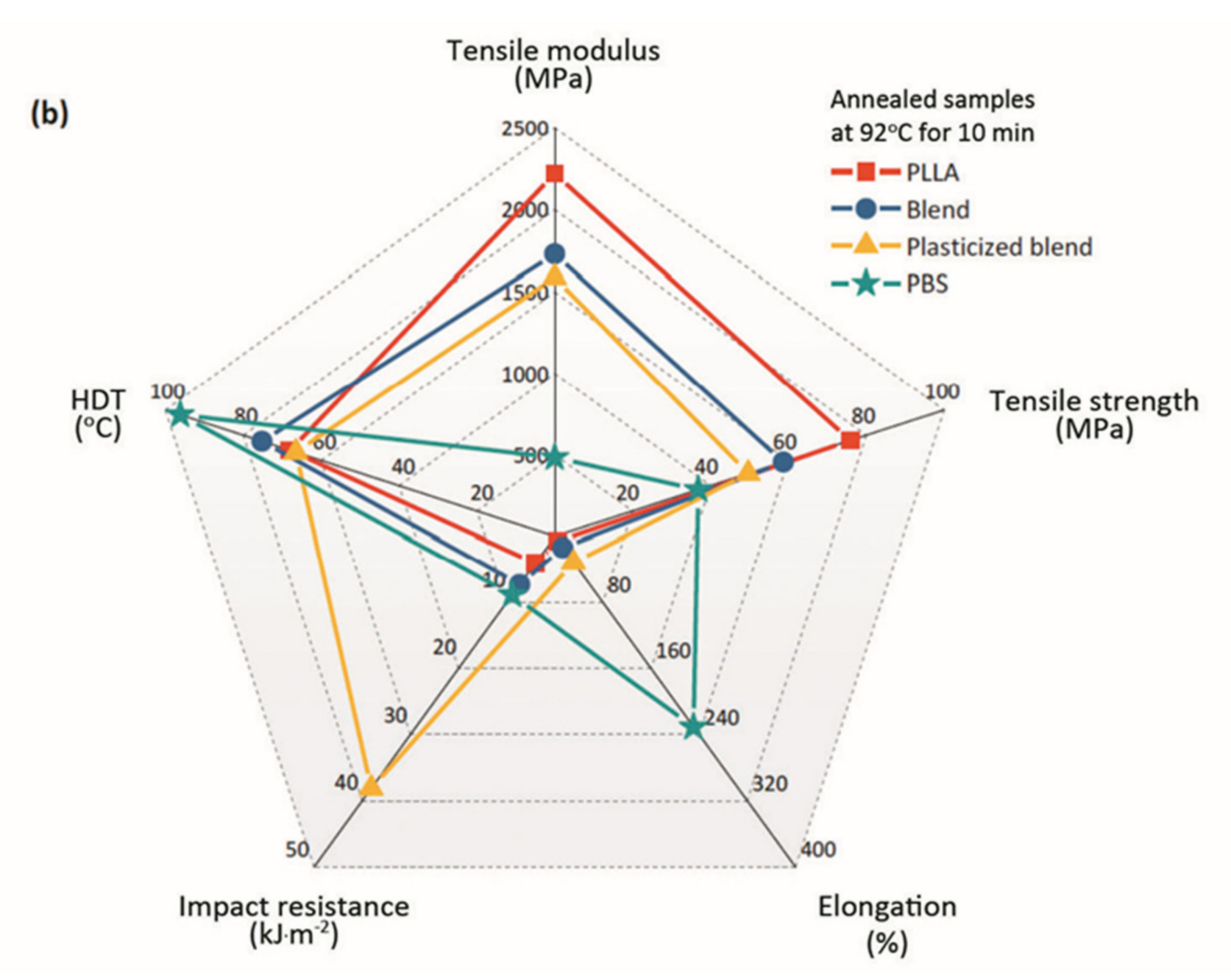
Overall performance of non-annealed samples (**a**), and of annealed samples (at 92 °C 10 min) (**b**).

The overall performance of the samples before and after heat-treatment given in Figure 6 demonstrates that the PLLA-based blends consisting of PBS and PEG could offer specific material properties. A short period of thermal annealing from solid-state (10 min) is efficient for concurrently enhancing the impact strength and heat stability of the PLLA-based materials. Biodegradable materials with balanced rigidity (modulus ~1.60 GPa), toughness (impact strength ~40 kJ·m^−2^), and thermal resistance (HDT ~67 °C) are successfully prepared by controlling the properties of polymers at the microscopic level through the concept of heat treatment. Although super-tough PLLA (impact strength > 53 kJ·m^−2^ or 530 J·m^−1^ as proposed by Wu) [39] could not be achieved in this study, the resulting impact is significant to substitute high-impact plastics such as acrylonitrile butadiene styrene (ABS) with the reported impact strengths around 16–21 kJ·m^−2^ [23,44]. In industry, PLLA and ABS are two of the most common materials used in FDM 3D printing technology. PLLA has advantages in terms of its bio-based, biodegradability, high stiffness, and low printing temperature. However, the brittleness of PLLA has greatly limited its use in producing certain industrial 3D printing products such as automotive parts; or medical products and equipment where high modulus, strength, and toughness are essential. ABS is often preferred due to its improved ductility and toughness over PLLA. The results of this study clearly demonstrate that the brittleness of PLLA can be overcome by manipulating the crystallization and morphology of PLLA via a combined melt-blending and thermal annealing approach. Hence, the production of fully biodegradable parts with comparable properties to ABS by FDM 3D printing is highly possible.

## 4. Conclusions

The PLLA-based blends consisting of PBS and medium molecular-weight PEG could offer specific material properties. The addition of 20% PBS increased the tensile elongation of PLLA significantly; however, it hardly improved the impact resistance. The PLLA/PBS blend cannot achieve the anticipated improvement in the impact properties by either adding PEG plasticizer or performing heat treatment. The resistance to impact load was gained only after the PLLA/PBS blends were plasticized with 5% PEG and simultaneously heat-treated under the appropriate conditions. The favorable annealing temperature for the PLLA-based blend was at 92 °C. In this regard, the plasticized blends after applying heat treatment for 10 min exhibited a high impact resistance of 38.4 kJ·m^−2^ which is nearly ten-fold over that of the starting PLLA, with a modulus of 1.60 GPa and HDT of 67 °C.

The results indicated that increasing crystallinity alone did not ensure the enhancement of impact toughness. Crystalline morphology, phase morphology, and compatibility in the system played a decisive role in determining the impact toughness of the blends. The microscopic study showed the fracture mechanism changes from crazing to shear yielding in the annealed-plasticized blend. The annealing temperatures had more effects on impact strength and tensile elongation than the modulus. The results of this study demonstrated that controlling the properties of polymers at the microscopic level through the concept of heat treatment is a promising strategy for obtaining high toughness PLLA of balanced stiffness and heat resistance.

## Figures and Tables

**Table 1:** Effect of annealing temperature on tensile modulus, strength, elongation at break, and impact strength of PLLA, PLLA/PBS blend, and plasticized blend. (Annealing time 10 min).

Sample	Annealing Temperature (°C)	Modulus (MPa)	Tensile Strength (MPa)	Elongation (%)	Impact Strength (kJ∙m^−2^)
PLLA	-	2045.24 ± 157.34	68.48 ± 1.64	12.60 ± 3.00	4.02 ± 0.17
	75	2197.41 ± 56.18	74.38 ± 2.14	6.48 ± 0.31	3.92 ± 0.06
	92	2218.89 ± 81.38	76.10 ± 1.76	6.69 ± 0.48	4.92 ± 0.48
	152	2402.50 ± 82.47	67.33 ± 4.72	5.15 ± 0.69	3.75 ± 0.13
Blend	-	1781.23 ± 118.90	55.69 ± 1.08	356.99 ± 14.11	5.85 ± 0.04
	75	1586.70 ± 58.20	56.03 ± 1.83	213.75 ± 69.96	6.92 ± 0.31
	92	1728.59 ± 55.20	59.11 ± 1.45	14.31 ± 2.84	8.13 ± 0.63
	152	1940.16 ± 97.20	55.80 ± 1.73	10.09 ± 2.35	5.27 ± 0.34
Plasticized blend	-	1655.07 ± 98.70	48.55 ± 0.55	380.77 ± 21.98	6.75 ± 0.53
	75	1665.07 ± 46.94	53.12 ± 1.81	52.04 ± 10.33	32.89 ± 1.64
	92	1587.34 ± 97.31	50.17 ± 1.31	32.04 ± 11.25	38.34 ± 2.22
	152	1893.70 ± 83.01	49.43 ± 1.75	18.98 ± 3.20	7.16 ± 1.16
